# Construction of 1D Vortex Chain Using a Chiral Nanostructure

**DOI:** 10.1002/advs.202001040

**Published:** 2020-07-01

**Authors:** Zhenghua Li, Bin Dong, Yangyang He, Xiang Li, Aiying Chen

**Affiliations:** ^1^ Key Laboratory of New Energy and Rare Earth Resource Utilization of State Ethnic Affairs Commission School of Physics and Materials Engineering Dalian Minzu University Dalian 116600 China; ^2^ School of Materials Science and Engineering University of Shanghai for Science and Technology Shanghai 200093 China

**Keywords:** chirality, magnetic force microscopy, magnetic vortex, spin waves (SWs)

## Abstract

The construction and control of high‐order coupled vortices are a significant challenge for promoting the application of magnetic vortices. Thus far, only double‐coupled vortices have been produced and modulated in some ferromagnetic nanostructures. Here, an effective approach is provided to obtain a high‐order coupled vortex structure by using a chiral nanostructure. Double‐vortex, triple‐vortex, and *n*‐vortex chains can be successfully constructed using structured Fe_4_N nanostrips and bias nanomagnets. The designed chiral nanostructure cannot only control the transport and hybridization of vortices but also modulate the domain walls of the vortex chain for spin wave (SW) propagation. At the exciting frequency of 1.2 GHz, the SW propagates along the domain walls formed in the vortex chain. Upon increasing the frequency to 5.0 GHz, the SW gradually spreads from the domain walls into domains. This technique will present a new perspective for the design and application of magnetic vortex‐based devices.

## Graphical abstract

An effective approach to obtain a 1D vortex chain by using a chiral nanostructure is provided. Double‐vortex, triple‐vortex, and *n*‐vortex chain are constructed using structured Fe_4_N nanostrips and bias nanomagnets. The spin wave propagates along the domain walls formed in the vortex chain at the frequency of 1.2 GHz, and spreads from the domain walls into domains at 5.0 GHz.

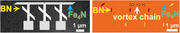

## 1 Introduction

Magnetic vortices occur when the magnetic spins “whirl” around a polarized core, and they have two conceivable states that depend on the direction of the spins: clockwise (CW) or anticlockwise (ACW).^[^ ^1^, ^2^, ^3^, ^4^, ^5^, ^6^, ^7^ ^]^ As a topologically protected structure that exists in ferromagnetic materials, the magnetic‐coupled vortex structure is significantly attractive, as it can simultaneously possess both CW and ACW vortices.^[^ ^8^, ^9^, ^10^, ^11^, ^12^, ^13^ ^]^

Compared with noncoupled vortices, coupled‐vortices offer obvious advantages in terms of data storage and spin propagation because of vortex–vortex coupling and strong magnetic dipolar interactions.^[^ ^1^, ^10^, ^11^, ^12^, ^14^, ^15^, ^16^, ^17^ ^]^ Furthermore, coupled vortices can be easily controlled via magnetic fields or electric currents and detected using microscopic techniques,^[^ ^1^, ^8^ ^]^ such as scanning transmission X‐ray microscopy and scanning nitrogen‐vacancy magnetometry.

Coupled vortices can be formed via direct exchange or dipolar interaction between two separated vortices in the nanostructure, and their configuration depends on the combination of chirality and polarity.^[^ ^18^, ^19^, ^20^, ^21^, ^22^, ^23^ ^]^ Recently, the formation of magnetic coupled vortices was investigated by introducing magnetic trilayers, planar heterostructures, magnetic nanodisks, among others in ferromagnetic nanostructures.^[^ ^1^, ^6^, ^9^, ^10^, ^12^, ^13^, ^14^, ^16^, ^17^ ^]^ However, thus far, only double‐coupled vortices have been produced and modulated. To promote the potential application of magnetic vortices, scientists have always intended to find methods to construct and control high‐order coupled vortex structures.

In this letter, we provide a novel and effective approach to obtain a high‐order coupled vortex structure (from double‐vortex to n‐vortex chain) with a chiral nanostructure. The designed chiral nanostructure cannot only control the transport and hybridization of vortices but also modulate the nanochannels for spin wave (SW) propagation. This technique will introduce a new perspective for the design and application of magnetic‐vortex‐based devices.

## 2 Results

### 2.1 Principle of Constructing the Vortex Chain

When a vortex pair (CW and ACW) interact with each other, a double‐vortex structure can be formed because of the direct exchange and dipolar interaction (Section S1, Supporting Information). Here, the CW‐vortex is defined as C and ACW‐vortex as A. The formation of a double‐vortex is described as follows:

(1) $$\begin{array}{c} C⊕A\to CA \end{array}$$

The term CA denotes the magnetic double‐vortex state. Using the same principle as that in Equation (1), a triple‐vortex state can also be formed as follows:

(2) $$\begin{array}{c} C⊕A⊕C\to CAC \end{array}$$

Using Equations (1) and (2), we can deduce the principle of constructing a vortex chain by using vortices with different chiralities, as follows:

(3) $$\begin{array}{c} \underset{2n}{\underset{︸}{C_{1}⊕A_{1}⊕\cdot \cdot \cdot \cdot \cdot \cdot C_{n}⊕A_{n}}}\to \underset{2n}{\underset{︸}{C_{1}A_{1}\cdot \cdot \cdot \cdot \cdot \cdot C_{n}A_{n}}} \end{array}$$

When a CW vortex interacts with another CW vortex, an ACW‐vortex core will be generated (Section S1, Supporting Information) as follows:

(4) $$\begin{array}{c} C⊗C\to CAC \end{array}$$

where CAC denotes the triple‐vortex state. On the basis of this principle, three CW vortices can form a five‐vortex structure as follows:

(5) $$\begin{array}{c} C⊗C⊗C\to CACAC \end{array}$$

The extension of Equation (5) gives the principle of constructing a vortex chain by using *n* vortices with the same chirality, as follows:

(6) $$\begin{array}{c} \underset{n}{\underset{︸}{C_{1}⊗C_{2}⊗\cdot \cdot \cdot \cdot \cdot \cdot ⊗C_{n}}}\to \underset{2n-1}{\underset{︸}{C_{1}A_{1}C_{2}\cdot \cdot \cdot \cdot \cdot \cdot A_{n-1}C_{n}}} \end{array}$$

From Equation (6), we can observe that the number of cores (*N*) in a vortex chain depends on the number of vortices (*n*), and that the formation of a 1D vortex chain by using *n* vortices with the same chirality obeys the following principle: *N* = 2*n* – 1 (*n* ≥ 2).

### 2.2 Chiral Control of Vortex

**Figure 1:**
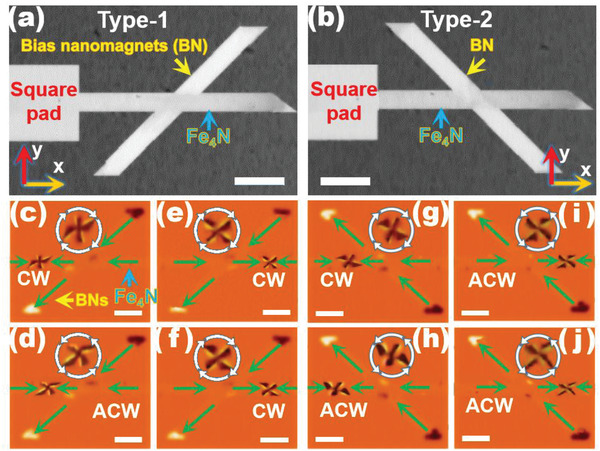
Chiral control of vortex. a,b) SEM of the Fe_4_N nanostrips and BNs. c,d) AF‐MFM of vortices (CW and ACW) injected into the Fe_4_N nanostrip for type‐1. e,f) AF‐MFM of the vortices (CW) passed through type‐1 BN. g,h) AF‐MFM of the vortices (CW and ACW) injected into the Fe_4_N nanostrip for type‐2. i,j) AF‐MFM of the vortices (ACW) passed through the type‐2 BN. The magnified vortex structures are shown as inserts in the AF‐MFM images. Green arrows indicate the direction of magnetization in the domains. The DC magnetic field is continuously applied during the AF‐MFM tests to maintain a static equilibrium state. The white color bars represent 750 nm.

The SEM images of the designed device are depicted in **Figure** **1**a,b. The magnetic properties of bias nanomagnet (BN) are presented in Section S2 (Supporting Information). The length and width of Fe_4_N nanostrips are 3.25 µm and 300 nm, respectively. Square pads with the side length of 1 µm are incorporated for injecting the vortex walls configurations. The angle between the Fe_4_N nanostrips and BNs is 45° (type‐1, Figure 1a) and 135° (type‐2, Figure 1b), respectively. The Fe_4_N nanostrips are full saturated via a magnetic field (H) of 1000 Oe along the ‐*x* axis for 5 s.

The vortices with CW or ACW chirality generated from the square pads are injected into the Fe_4_N nanostrips via a DC field of 75 Oe along the +*x* axis, as depicted in Figure 1c,d for type‐1, and Figure 1g,h for type‐2, as confirmed via alternating force magnetic force microscopy (AF‐MFM).^[^ ^24^, ^25^ ^]^ The driven field of 75 Oe is well beyond the Walker breakdown field of the Fe_4_N nanostrip (Section S3, Supporting Information). Upon increasing H to 150 Oe, the vortices pass through BNs, as depicted in Figure 1e,f for type‐1, and Figure 1i,j for type‐2. In type‐1 (see Figure 1e,f), only CW chirality is detected when vortices (CW and ACW) pass through type‐1 BN. Contrarily, only ACW chirality is probed when vortices (CW and ACW) pass through type‐2 BN (see Figure 1i,j). During the AF‐MFM tests, the DC magnetic field is continuously applied to maintain a static equilibrium state. From the results, it is proved that the chiral control of vortex can be realized by using the designed nanostructure, which is a nonvertical nanocross of Fe_4_N nanostrip and BN. A good repeatability of this chiral response is confirmed with the same experimental conditions for more than 50 times. Other cross‐type nanostructures (crossing angles of 30°, 60°, and 90°) are presented in Section S4 (Supporting Information). The determination of vortex chirality via AF‐MFM is depicted in Section S5 (Supporting Information).

### 2.3 Construction of Coupled Vortices

**Figure 2:**
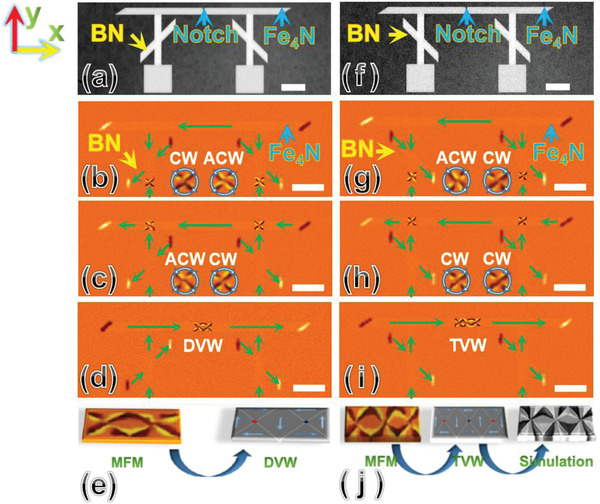
Construction of a coupled vortex. a) The SEM of Fe_4_N nanostrips and BNs with the angles of 45° and 135°. b) The AF‐MFM of the vortex pair (CW and ACW) injected into the Fe_4_N nanostrips. c) The vortex pair (ACW and CW) passed through the BNs. d) The ACW–CW vortex pair converges and forms a DVW . e) The DVW structure with one ACW core and one CW core. f) The SEM of the Fe_4_N nanostrips and BNs with the angle of 45°. g) The vortex pair (ACW and CW) injected into the Fe_4_N nanostrips. h) The vortex pair (CW and CW) passed through the BNs. i) The CW–CW vortex pair converges and forms a TVW . e) The TVW structure with one ACW core at center and j) two CW cores at both sides. The magnified vortex structures are shown as inserts in the AF‐MFM images. Green arrows indicate the direction of magnetization in the domains. The white color bars represent 1 µm.

In **Figure** **2**a, we depict the SEM image of the designed nanostructure with a couple of BNs and square pads. The crossing angles of these two nanocrosses are set to be 45° and 135°, respectively. A notch is introduced at the center of the Fe_4_N nanostrip for pinning the domain wall (the detection of pinning sides along the Fe_4_N nanostrip is presented in Section S6, Supporting Information). The Fe_4_N nanostrip is full saturated via a magnetic field (H) of 1000 Oe along the ‐*y* axis for 5 s. Subsequently, the H field along the +*y* axis is configured in the following sequence: 75 Oe→150 Oe→0 Oe. The AF‐MFM images and schematic of the vortex distribution are depicted in Figure 2b–e. When H is 75 Oe during the measurements, a vortex pair (CW and ACW) generated from the two square pads is injected into the Fe_4_N nanostrips, as depicted in Figure 2b. In fact, the initial vortex pair generated from the square pads are randomly configured, thus, it is possible to generate CW–ACW, ACW–CW, or ACW–ACW vortex pair etc (Section S7, Supporting Information). As depicted in Figure 2c, the vortex pair passes through BNs and is then relaxed at the top edges upon increasing H to 150 Oe for 5 s (the magnetic process of the vortex pair is confirmed by micromagnetics, Section S8 (Supporting Information); the stability of vortices is confirmed by AF‐MFM, Section S9, Supporting Information). Notably, both the vortices reverse their chirality after passing through the BNs. Next, when H is gradually increased to 140 Oe along the +*x* axis during the measurements, the ACW and CW vortices interact with each other via magnetic exchange and dipolar interaction, and they converge to form a double‐vortex‐wall (DVW) structure (Section S1, Supporting Information), as depicted in Figure 2d. After removing the magnetic field, the DVW structure keeps stable when the H field is reduced to zero (Section S10, Supporting Information). In Figure 2e, we depict the DVW structure in the Fe_4_N nanostrip.

In Figure 2f, we depict the condition of the device including two same crossing angles each of 45°. The experimental procedure is similar to that in Figure 2b–d. From Figure 2g, it is evident that a vortex pair (ACW and CW) generated from the two square pads is injected into the Fe_4_N nanostrips. However, only the right vortex (i.e., CW) conserves its chirality, while the left one (i.e., ACW) reverses to CW after passing through the BN (see Figure 2h). Upon gradually increasing H to 140 Oe along the +*x* axis during the MFM tests, the CW–CW vortex pair converges near the notch. Because both the vortices in the CW–CW vortex pair possess the same chirality, they are coupled via the generation of a new vortex core for energy minimization (Section S11, Supporting Information). Finally, an equilibrium topological structure is generated accompanied by the change of magnetic poles of the nanostrip (Section S11, Supporting Information), as depicted in Figure 2i. On the basis of micromagnetic simulation, the CW–CW vortex pair converges and forms a triple‐vortex‐wall (TVW), which includes three vortices with one ACW core in the center and two CW cores on both sides (see Figure 2j) and has been confirmed as a stable topological structure in magnetic thin films.^[^ ^26^ ^]^ The stability of TVW under zero magnetic field is verified by AF‐MFM and micromagnetics (Section S12, Supporting Information).

### 2.4 Construction of Vortex Chains

**Figure 3:**
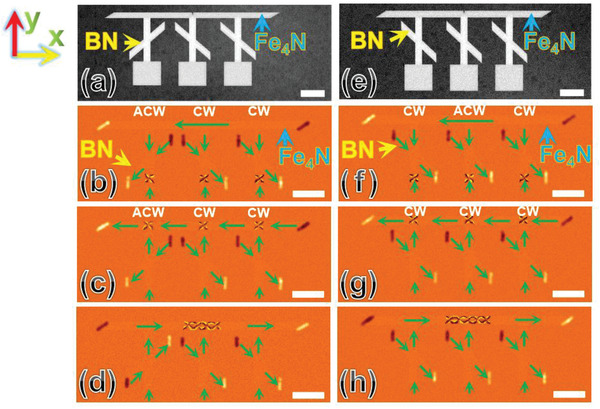
Construction of vortex chains. a) The SEM image of the designed device with three square pads and BNs; the crossing angle of Fe_4_N nanostrips and BNs are 135°, 45°, and 45°, respectively. b) The AF‐MFM of vortices (ACW, CW, and CW) injected into the Fe_4_N nanostrips. c) The vortices (ACW, CW, and CW) passed through BNs. d) Vortices converge and form a four‐vortex chain. e) The SEM image of the designed device with three square pads and BNs; the crossing angles of the Fe_4_N nanostrips and BNs are 45°. f) Three vortices (CW, ACW, and CW) injected into the Fe_4_N nanostrips. g) Three vortices (CW) passed through the BNs. h) Three CW‐vortices converge to form a five‐vortex chain. Green arrows indicate the direction of magnetization in the domains. The white color bars represent 1 µm.

In **Figure** **3**a, we depict the SEM image of the designed device with three square pads and BNs; the crossing angle of Fe_4_N nanostrips and BNs is 135°, 45°, and 45°, respectively. The Fe_4_N nanostrip is full saturated via a magnetic field (H) of 1000 Oe along the ‐*y* axis for 5 s. The AF‐MFM images of the vortex distribution on the nanostructure are depicted in Figure 3b–d. When H is 75 Oe along the +*y* axis during the measurements, three vortices (ACW, CW, and CW) are simultaneously injected into the Fe_4_N nanostrips, as depicted in Figure 3b. In Figure 3c, three vortices pass through the BNs on the basis of the chirality rectification procedure upon increasing H to 150 Oe along the +*y* axis for 5 s. Finally, a four‐vortex chain can be constructed in the Fe_4_N nanostrip when H is gradually increased to 150 Oe along the +*x* axis during the MFM tests, as depicted in Figure 3d.

In Figure 3e, we depict the condition of the device for three same crossing angles of 45°. The experimental conditions are similar to that in Figure 3b–d. From Figure 3f, it is evident that the three vortices (CW, ACW, and CW) generated from the pads are injected into the Fe_4_N nanostrips. However, only the center vortex (ACW) reverses to CW after passing through the BN (see Figure 3g). The three CW‐vortices converge to form a five‐vortex chain accompanied by the change of magnetic poles of the nanostrip (Section S13, Supporting Information), as depicted in Figure 3h. On the basis of the results, we observe that the number of cores (*N*) in the vortex chain depends on the number of square pads (*n*), and that the formation of a 1D vortex chain by vortices with the same chirality obeys the following principle: *N* = 2*n*– 1 (*n* ≥ 2).

### 2.5 Quasistatic Mechanism of Vortex Chain

**Figure 4:**
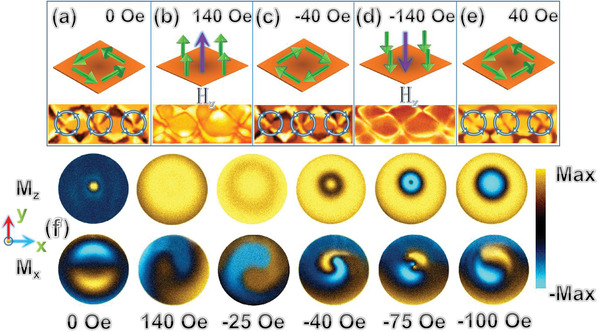
a–e) AF‐MFM images of quasi‐static magnetization reversal and chirality switching of vortex chain. f) Micromagnetics simulation of the vortex‐reversal mechanism.

**Figure 5:**
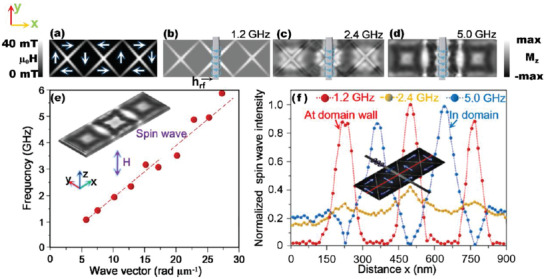
Vortex chain for SW propagation. a) The absolute value of the demagnetizing field of vortex chain; the arrows indicate the directions of the magnetic moments. b–d) The perpendicular component of the magnetization distribution (M*_z_*) of vortex excited by the microwave field at the frequencies of 1.2, 2.4, and 5.0 GHz. e) The calculated dispersion relations of SW in the vortex chain. f) The SW spectra excited by the microwave field (from 1.2 to 5.0 GHz) along the central axis (from 0 to 900 nm) of the vortex chain; a schematic of the Fe_4_N nanostrip incorporated with a nano‐antenna is inserted.

The quasistatic magnetization reversals of the vortex chain are studied via AF‐MFM,^[^ ^24^, ^25^ ^]^ as depicted in **Figure** **4**a–e. The vortex chain is induced using an H‐field (Hz) that is perpendicular to the sample surface, and Hz is configured in the following sequence: 0 Oe→140 Oe→–40 Oe→ –140 Oe→40 Oe. In Figure 4a,b, we depict the snapshots of the vortex chain in the first reversal process (i.e., 0 to 140 Oe; Hz along the +*z* axis). At this stage, the magnetization gradually rotates from in‐plane (0 Oe) to the +*z* axis (140 Oe); meanwhile, the chiralities of the vortices are conserved. In the second reversal process as Hz is reduced from 140 to ‐40 Oe (see Figure 4c), the chiralities of the vortices simultaneously change because of the rotation of magnetization from the +*z* axis to in‐plane. Subsequently, the magnetization rotates from the in‐plane to ‐*z* axis as Hz is changed from –40 to –140 Oe (see Figure 4d). In Figure 4e, we depict the snapshot of the vortex chain in the subsequent reversal process (i.e., when Hz shifts from ‐140 to 40 Oe); the vortices reverse again, and their chiralities simultaneously change. The quasistatic reversal of the vortex chain indicates that the chiralities of the vortices periodically change in the presence of a magnetic field, and that the magnetic moments of the vortices also reverse with periodicity because of the strong magnetic exchange and dipolar interactions. In **Figure** **5**f, we depict the switching mechanism of a vortex via micromagnetics. When H‐field points to the +*z* axis during the first reversal stage (i.e., 0 to 140 Oe), the vortex core expands to cover the entire domain region; during this process, the vortex chirality is conserved. When H‐field shifts to the –*z* axis in the subsequent reversal process, the spins near the edge first reverse and will shrink toward the center of the vortex core (–25 to –40 Oe); meanwhile, the chirality of the vortex changes (–40 Oe). Subsequently, the vortex core reverses to the –*z* axis (–75 to –100 Oe) and expands again to cover the entire domain region. The simulation results can be used to explain the switching mechanism of vortex chain. From the results, it is confirmed that the vortex chain is dynamically stable and can be easily reversed via magnetic fields, thereby introducing a new perspective in the domain of magnetic‐data storage.

### 2.6 Vortex Chain for SW Propagation

The potential application of vortex chain for SW propagation is depicted in Figure 5a–f. In Figure 5a, we depict a micromagnetic model of the chain structure. The arrows indicate the magnetization distribution, and the white–black color denotes the distribution of effective magnetic fields. The result shows large magnetic fields at domain walls due to the accumulation of magnetic charges near the domain walls.

An oscillating magnetic field *h*_rf_ (*h*_rf_ = *h*_0_sin(*ωt*)) generated by microwave current is modeled for the local excitation of spin waves. The excitation frequency *f* = *ω*/2*π* is varied between 1 and 5 GHz. The amplitude of the oscillating magnetic field is around 50 Oe. The response of the vortex chain to an oscillating magnetic field *h*_rf_ is calculated at three various frequencies. In Figure 5b–d, we depict the calculated normalized *z*‐component magnetization (M*_z_*) a certain time once a stable oscillation is achieved. The exciting frequency is fixed at 1.2, 2.4, and 5.0 GHz, respectively. At the frequency of 1.2 GHz, the SW propagates along the domain walls because of the formation of potential wells at the domain walls. Upon increasing the frequency to 2.4 GHz, the SW gradually spreads from the domain walls into domains. Upon increasing the frequency to 5.0 GHz, the SW is confined in the domain. In Figure 5e, we depict the calculated dispersion results of the SW in vortex chain, and a nearly linear relation between frequency and wave vector is shown, indicating that the magnetic dipolar interaction dominates the spin dynamics. The SW excited in a single vortex structure is also presented in Section S14 (Supporting Information).

In Figure 5f, we depict the SW spectra of the vortex chain detected via near‐field Brillouin light scattering spectroscopy (BSL) incorporated with an atomic force microscopy (Section S15, Supporting Information). A nanoantenna is incorporated to excite the SW of vortex chain. The details of the nanoantennae are shown in Section S16 (Supporting Information). The vortex chain is excited by the oscillating magnetic field *h*_rf_ generated by microwave current (*I*_rf_ = *I*_0_sin(*ωt*)), the excitation frequency *f* = *ω*/2*π* is varied between 1 and 5 GHz with amplitudes of the current around 1 mA. The microwave current is continuously applied during the BSL measurement. A schematic of the measured vortex chain structure is inserted. The SW spectra of vortex chain are detected along the central axis of the vortex chain (marked using red line) at the microwave frequencies of 1.2, 2.4, and 5.0 GHz, respectively. At the frequency of 1.2 GHz, the SW signal is mainly confined near the domain walls. The SW gradually transfers from the domain walls into domains at 2.4 GHz, but their SW signal intensities are weak. Upon increasing the frequency to 5 GHz, the SW signals spread into domains, and the intensity is enhanced again. It is revealed that the SW signal is mainly confined near the domain walls of vortex chain at low frequency (1.2 GHz). An obvious trend is that the confinement of SW at the domain walls gradually weakens upon increasing the frequency, agreeing well with the results of Figure 5b–d) and confirming that the vortex chain can realize SW propagation along domain walls at certain frequencies.

## 3 Discussion

The chiral control of vortex by the angled BN accords with the conservation of topological defects, the analysis of the winding number is presented in Section S17 (Supporting Information). The selectivity between CW and ACW is explained as the mechanism of chiral rectification and theoretically demonstrated using a micromagnetic model of vortex domain walls (Sections S18, Supporting Information). As a single vortex approached the BN, if the spins near the guiding edge of the vortex oriented parallel to the magnetization inside the BN, the vortex can pass through the barrier and conserved its chirality for energy minimization. Contrarily, the chirality will reverse after the vortex passes through the barrier to avoid magnetic charge accumulation (Section S18, Supporting Information). This principle is of potential importance for spin‐based devices in which the chirality of vortex should be switched as it propagates through magnetic nanostrips. Additionally, this technique facilitates the design of complex vortex topologies and clarifies the mechanisms of coupled‐vortex reversals.

The formation mechanism of coupled vortices is studied using micromagnetics (Section S1, Supporting Information). A vortex pair (CW and ACW) is simultaneously generated using the square pads and injected into the magnetic nanostrip. When the CW and ACW vortices of the pair approach each other and interact, the vortex pair is weakly coupled through a single‐domain for decreasing the demagnetized energy. Upon further increasing the external fields, the single‐domain disappears because of the magnetic exchange and dipolar interaction. Finally, the CW–ACW vortex pair directly converges to form a DVW structure. However when the CW and CW vortices of a vortex pair interact with each other, the CW–CW vortex pair is coupled because of the generation of a new vortex core for energy minimization. Therefore, the CW–CW vortex pair converges and forms a TVW, which includes three vortices with a new ACW core in the center and two CW cores on both sides. On the basis of the results, we further successfully develop a technique to construct a 1D vortex chain. The number of cores (*N)* in the vortex chain depends on the number of square pads (n), and the formation of 1D vortex chain by using vortices with the same chirality obeys the following principle: *N* = 2*n* – 1 (*n* ≥ 2).

Finally, we theoretically and experimentally studied the intrinsic SW modes that propagate in vortex chain, as shown in the micromagnetic and BLS results of Figure 5. Notably, the SW is localized at the domain walls of the vortex chain at low frequency. Upon increasing the frequency to 5 GHz, the SW gradually spreads from domain walls into domains. Guiding the SW at the domain walls offers obvious advantages (short spin wavelength, high energy efficiency, among others) compared with it propagates in domains. Therefore, the SW is expected to propagate along the domain walls of vortex chain at a wide range of frequency. Generally, the SW modes transfer to higher frequency when the sizes of devices are reduced; therefore, the guiding of SW in domain walls shifts to higher frequency when the size of devices is further reduced.

## 4 Conclusion

This original result provided an easy approach to construct vortex chain by using a chiral nanostructure. Double‐vortex, triple‐vortex, and *n*‐vortex chain were successfully constructed using the structured Fe_4_N nanostrip and BN. In addition, the formation of a 1D vortex chain by using vortices with the same chirality obeys the following principle: *N* = 2*n* ‐ 1 (*n* ≥ 2). The constructed vortex chain can realize SW propagation along domain walls, as well as control the SW modes via exciting frequency. At the frequency of 1.2 GHz, the SW propagates along the domain walls because of the formation of potential wells. Upon increasing the frequency to 5.0 GHz, the SW gradually spreads from the domain walls into domains. This discovered mechanism introduces new perspectives for vortex‐based devices.

## 5 Experimental Section

##### Fabrication of Fe_4_N Nanostrip

The Ti (2 nm)/Fe_4_N(30 nm) films were deposited on (001)‐oriented SrTiO_3_ substrate at 420 °C using MBE technique. During the film growth, the deposition rate of Fe was set to 0.9 nm per minute, and the power of N_2_ plasma was fixed at 100 W, and then a Ti film was deposited to protect the Fe_4_N layer without oxidizing when exposed to air. The Fe_4_N nanostrips were prepared by electron beam lithography, lift‐off as well as reactive ions etching methods.^[^ ^27^ ^]^

##### Magnetic Characterization

AF‐MFM was utilized to determine the vortex chirality as well as the magnetic configurations with ultrahigh resolution, the imaging technique of AF‐MFM is described in Section S19 (Supporting Information). The BLS spectroscopy was employed to characterize the SW propagations in Fe_4_N nanostrip. Micromagnetics were used to investigate the vortex mechanisms as well as the SW dynamics based on Landau–Lifshitz–Gilbert equations (Section S20, Supporting Information).

## Conflict of Interest

The authors declare no conflict of interest.

## Author Contributions

Z.L. and B.D. designed the project. Z.L. and X.L. conducted AF‐MFM experiment, BSL and AMR measurement. Z.L. conducted SEM experiment. Z.L. and A.C. established the micromagnetic model. Z.L. and B.D. analyzed the data and wrote the paper. All authors contributed to the discussion of the results.

## Supporting information

Supporting Information
