# Supplementary material for: Construction of 1D Vortex Chain Using a Chiral Nanostructure
Source: Adv Sci (Weinh). 2020 Jul 1;7(16):2001040. doi: 10.1002/advs.202001040 (PMC7435250; doi:10.1002/advs.202001040)
Supplement: Supplementary file 1 — Supporting Information [file ADVS-7-2001040-s001.pdf]

Construction of one-dimensional vortex chain using a chiral nanostructure

Zhenghua Li[1], Bin Dong[1*], Yangyang, He[1], Xiang Li[2*], Aiying Chen[2]

[1]Key Laboratory of New Energy and Rare Earth Resource Utilization of State Ethnic Affairs Commission, School of Physics and Materials Engineering, Dalian Minzu University, Dalian, 116600, China

[2]School of Materials Science and Engineering, University of Shanghai for Science and Technology, Shanghai, 200093, China

[*]**Corresponding authors**: dong@dlnu.edu.cn, xiangli@usst.edu.cn

Section 1: The formation mechanism of higher-order coupled-vortex

Figure S1(a)-(d) show the formation mechanism of a double-vortex-wall (DVW) by micromagnetics. The Fe_4N nanostrip is initially magnetized by a magnetic field (H) of 1000 Oe along -x axis, and then H field along +x axis is configured as the following sequences: 0 Oe→100 Oe→120 Oe→140 Oe. When H=0 Oe, vortex pair (CW and ACW) are simultaneously formed inside the square pads, as shown in Figure S1(a). With the increase of H to 100 Oe along +x axis, the cores of the vortex pair move from the square pads to Fe_4N nanostrip, as shown in Figure S1(b), suggesting that the magnetic reversal begins by domain wall motion. When H is further increased to 120 Oe along +x axis, the CW-ACW vortex pair approach one another and interact with each other, it is observed that vortex pair are weakly coupled through a single-domain (marked by green square) for decreasing the demagnetized energy, as shown in Figure S1(c). Further increasing H to 140 Oe, the single-domain disappears due to the direct exchange and dipolar interaction, and the CW-ACW vortex pair directly converge to form a DVW structure (Figure S1(d)).

Figure S1(e)-(h) show the formation mechanism of a triple-vortex-wall (TVW) by using the same field sequence. Figure S1 (e) and (f) shows that vortex pair (CW

and CW) are simultaneously generated from the two square pads and injected into Fe_4N nanostrip, similar as the results in Figure S1 (a) and (b). However, when H is increased to 120 Oe, the CW-CW vortex pair are coupled through the generation of a new vortex core at bottom edge (marked by red circle) for energy minimization, as shown in Figure S1(g). Further increasing H to 140 Oe, CW-CW vortex pair converge to form a TVW, including three vortices with a ACW core in the center and two CW cores on both sides (shown in Figure S1(h)).

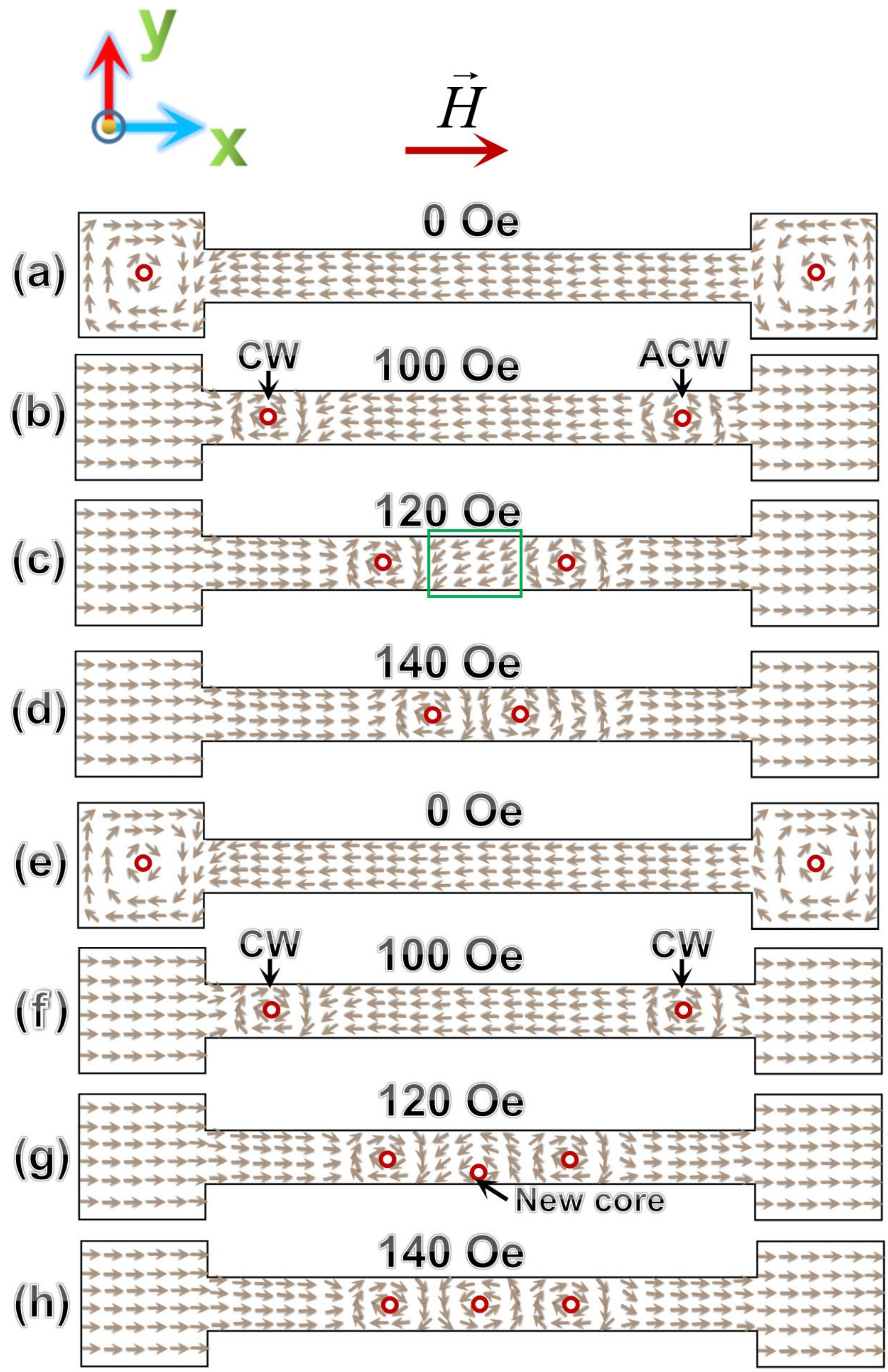

Figure S1. (a-h) the formation mechanism of higher-order coupled-vortex by micromagnetics.

Section 2 : The magnetic properties of BN

Figure S2(a) shows the SEM image of patterned BNs. The area of each BN is 600 × 6000 nm^2. The distance between neighboring BNs is more than 2 μm, which is far enough to neglect the magnetic dipole interactions. Figure S2(b) presents the AF-MFM image of BNs magnetized by a magnetic field (H) of 1000 Oe along +x axis (magnetic hard axis) for 5 second. Due to the geometrically-induced shape anisotropy, all BNs exhibit the feature of dark-bright dipole MFM contrast, indicating the formation of single domains with down or up orientations. Figure S2(c) and (d) present the simulated magnetic configuration and calculated MFM image of BNs magnetized by H of 1000 Oe along +x axis. The magnetization direction of BNs is parallel or anti-parallel to the shape-induced easy axis, as seen in Figure S2(c). The calculated MFM image (Figure S2 (d)) agrees well with the measured AF-MFM image (Figure S2(b)).

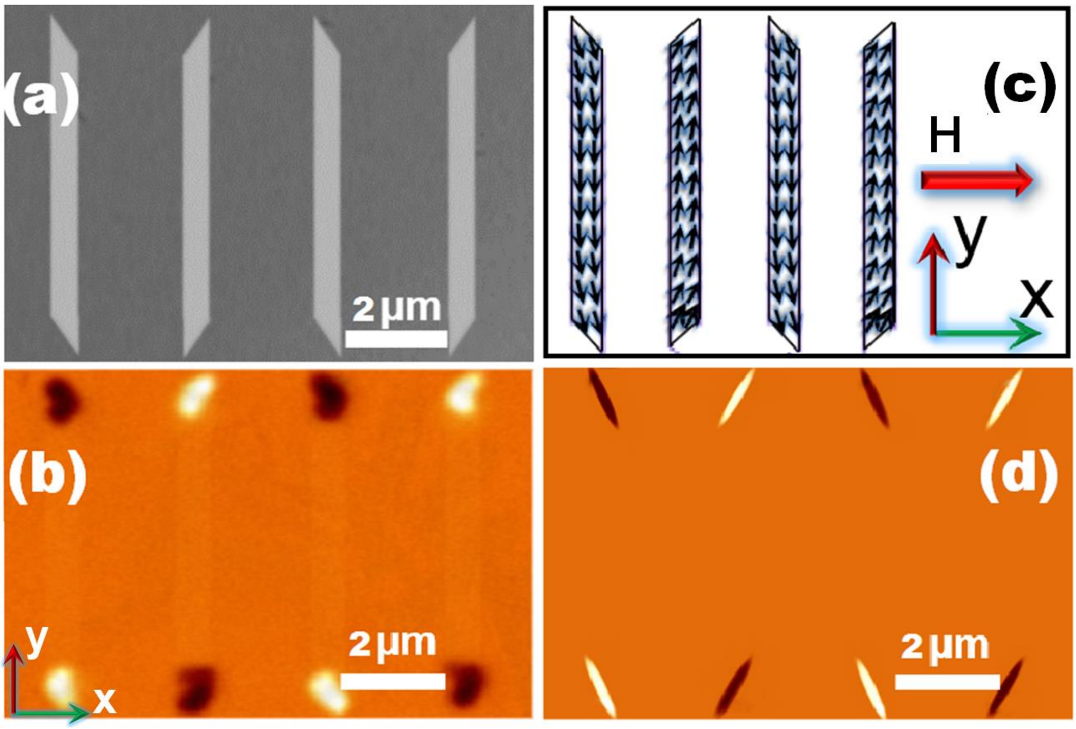

Figure S2. (a) SEM of BNs (b) AF-MFM of BNs in remanence. (c,d) Simulated magnetic configuration and calculated MFM image of BNs in remanence.

Section 3: The Walker breakdown field of the Fe_4N nanostrip

Figure S3 shows the vortex wall velocity as a function of the magnetic field (H)

for Fe_4N nanostrip with width of 300 nm and thickness of 30 nm. There is no notch and BN in this micromagnetic simulation. Dashed vertical line marks the Walker breakdown field. Symbols are results of numerical simulations.The simulated Walker breakdown field of the Fe_4N nanostrip is close to 15 Oe.

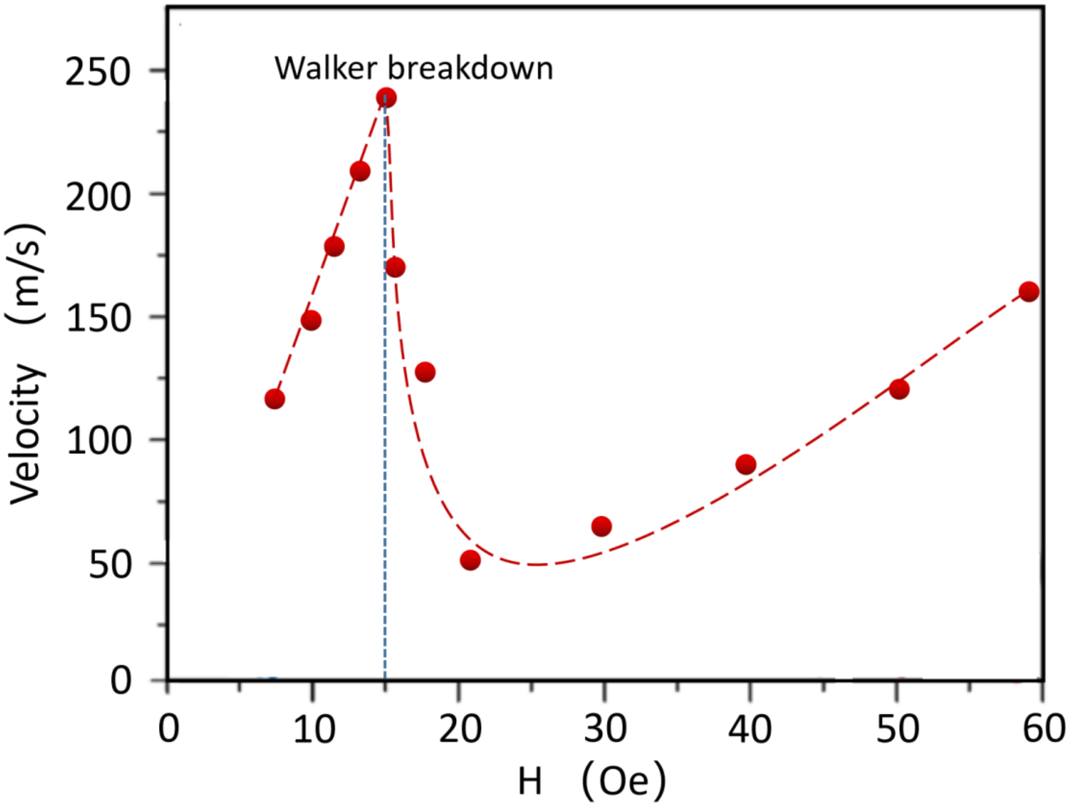

Figure S3. Vortex wall velocity as a function of the magnetic field (H) for Fe_4N nanostrip

Section 4: AF-MFM images of other cross-type nanostructures

Figure S4 shows the AF-MFM images of the cross-type nanostructures with the angle of 30°, 60° and 90°, respectively. In the case of 30° and 60°, the chiral control of vortex can be realized by the similar experimental procedure of Figure 1 in the text. However, the chiral rectifying of vortex can not be achieved with the cross angle of 90°, because when a vortex wall propagates through the BN, interaction with BN leads to the transformation of the domain wall from vortex to transverse wall (TW).

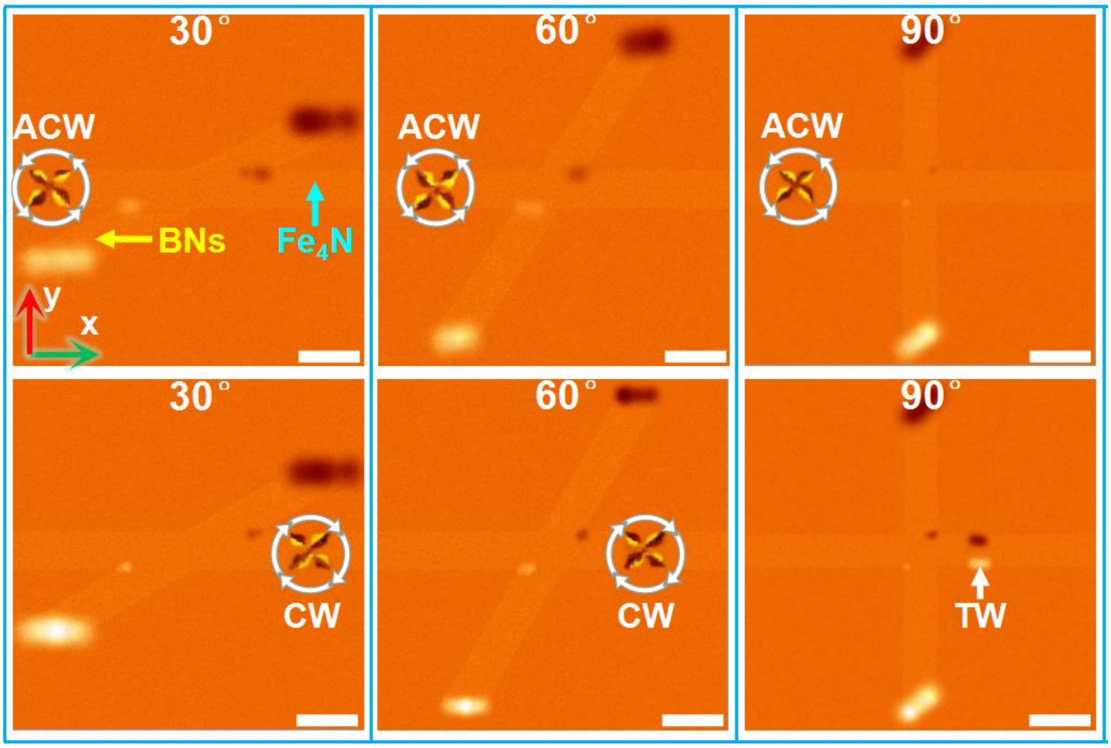

Figure S4. AF-MFM images of the cross-type nanostructures with the angle of 30°, 60° and 90°, respectively. The while colour bars represent 500 nm.

Section 5: AF-MFM images of FeCo disk, square pad and triangle dot

The AF-MFM images of FeCo disk, triangle and square dots are shown in Figure S5. For magnetic vortices with in-plane oriented spins, the AF-MFM only senses magnetic field that originated from the domain walls rather than the domain themselves. In the case of FeCo circular disk, no domain wall exists inside the circular-shaped vortex, and the AF-MFM result only presents the polarity of the vortex core. However, for square or triangle shape, domain walls form inside vortex due to the breaking of circular symmetry and generating of demagnetization field from square or triangle corners, therefore, the AF-MFM images present the distribution of magnetic field originated from the domain walls. The chirality of vortex can be identified by the direction of magnetic dipoles, as marked by the arrows (from dark to bright) in Figure S5.

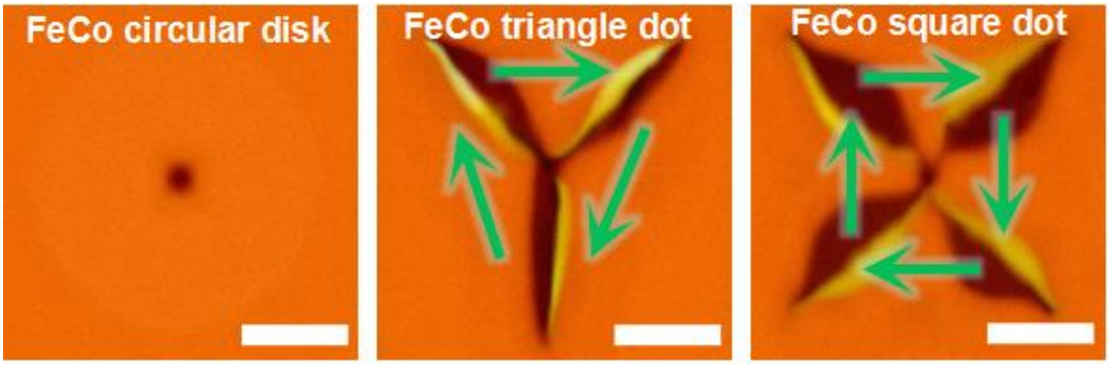

Figure S5. AF-MFM images of FeCo disk, triangle and square dots. The while colour bars represent 100 nm. The green arrows indicate the magnetization directions.

Section 6: Detection of pinning sites in Fe_4N nanostrip

In Figure S6, the Fe_4N nanostrip is initially magnetized by H of 1000 Oe along +x axis for 5 second. When H is 75 Oe along -x axis, a vortex domain wall generated from the pad is injected into the Fe_4N nanostrip. Afterwords, the AF-MFM imaging is performed on the nanostrip with zero applied field. The pinning sites give rise to the MFM contrast, with very weak bright/dark spots along the wire axis.

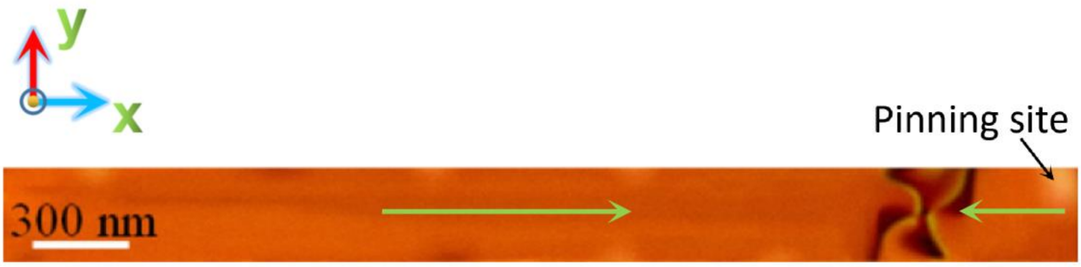

Figure S6. Detection of pinning sites in Fe_4N nanostrip by AF-MFM.The green arrows indicate the magnetization directions.

Section 7: Injection of CW-ACW, ACW-CW and ACW-ACW vortex pair by square pads

The initial vortex pair generated from the square pads are random, therefore, it is possible to generate CW-ACW, ACW-CW and ACW-ACW vortex pair etc. For 45° and 135° cross-type nanostructure (Figure 2 (a) in main text), the injection of CW-ACW, ACW-CW and ACW-ACW vortex pair is demonstrated (Figure S7 (a)-(c)). The experimental procedure is similar as Figure 2 (b) in the main text. When H is 75 Oe along +y axis, the CW-ACW, ACW-CW and ACW-ACW vortex pair generated from the square pads are injected into Fe_4N nanostrips before BNs (Figure S7 (a)-(c)).

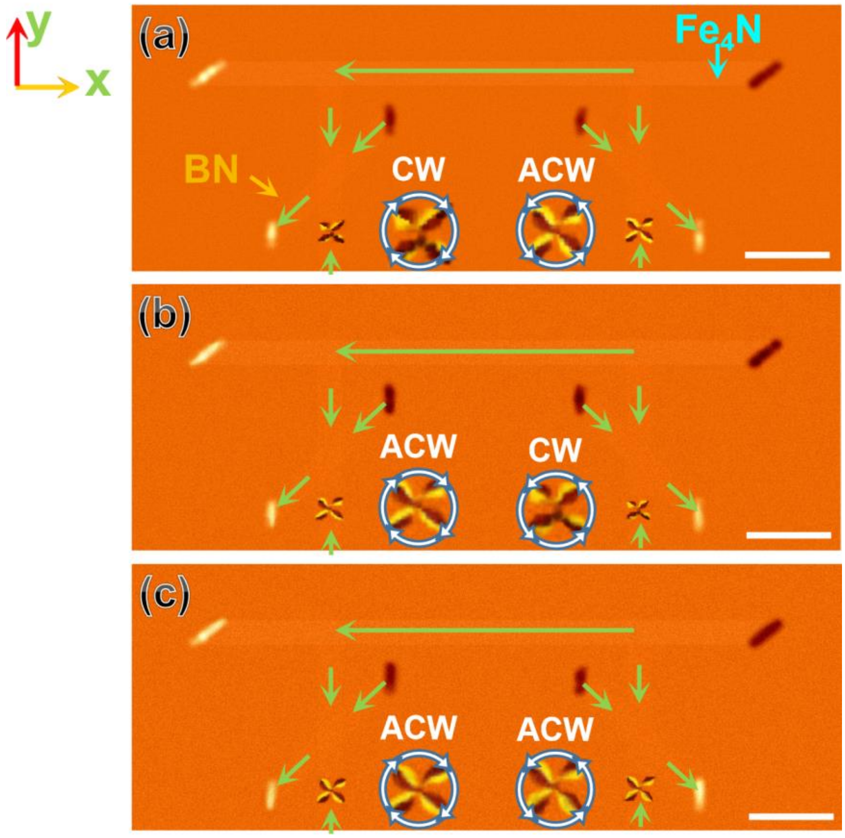

Figure S7. Injection of CW-ACW, ACW-CW or ACW-ACW vortex pair. The white colour bars represent 1 µm. The green arrows indicate the magnetization directions.

Section 8: The magnetization reversal of the device by applying a perpendicular magnetic field

The magnetization reversal of the device (Figure 2 (a)) with the increase of applied field from 0 to 150 Oe along +y axis is shown in Figure S8. The device is initially magnetized by H of 1000 Oe along -y axis. When H is 0 Oe, vortex pair (CW and ACW) are simultaneously generated inside the two square pads, as seen in Figure S8 (a). With the increase of H to 75 Oe along +y axis, the vortex pair are injected into Fe_4N nanostrips, as shown in Figure S8 (b). When further increasing H to 150 Oe along +y axis, the vortex pair move upward and park at the top edges, seen in Figure S8 (c). The magnetic field is continuously applied during the above process to maintain a static equilibrium state, it can be seen that the magnetization reversal governs by the domain walls motion. After removing the magnetic field, the vortex pair (CW and ACW) is relaxed near the top edges when the H field is reduced to zero, meanwhile, a new vortex pair is generated inside the square pads for energy minimization (Figure S8 (d)). Subsequently, when gradually increasing the applied field H from 0 to 140 Oe along the +x aixs, the vortex pair (CW and ACW) will converge to form a DVW structure near the notch.

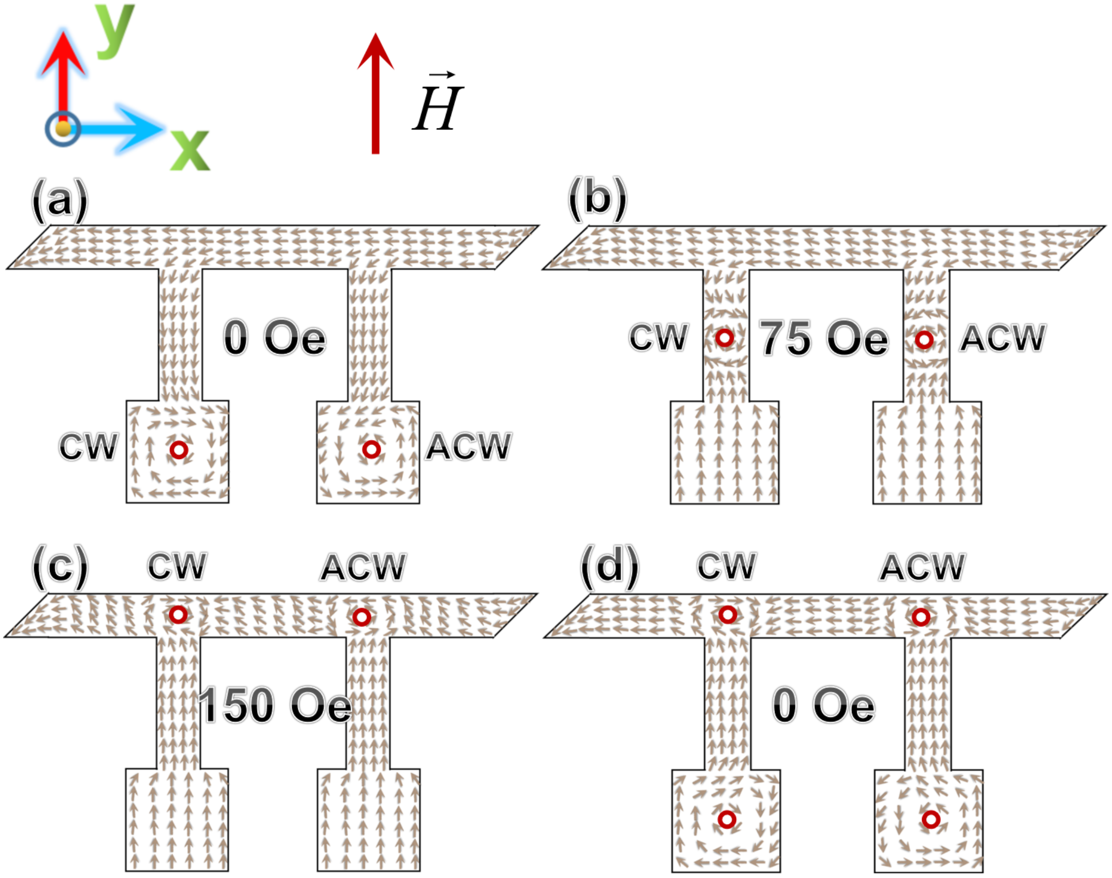

Figure S8. The magnetization reversal of the device with the increase of H along +y axis.

Section 9: Verifying the stability of vortices

The experimental procedure is similar as Figure 2 (b)-(c) in the main text, and then H is shifted to 50 Oe along -x axis, the AF-MFM image of vortices is shown in Figure S9, It can be seen that both vortices are stable. The white colour bars represent 1 μm, the green arrows represent the magnetization directions.

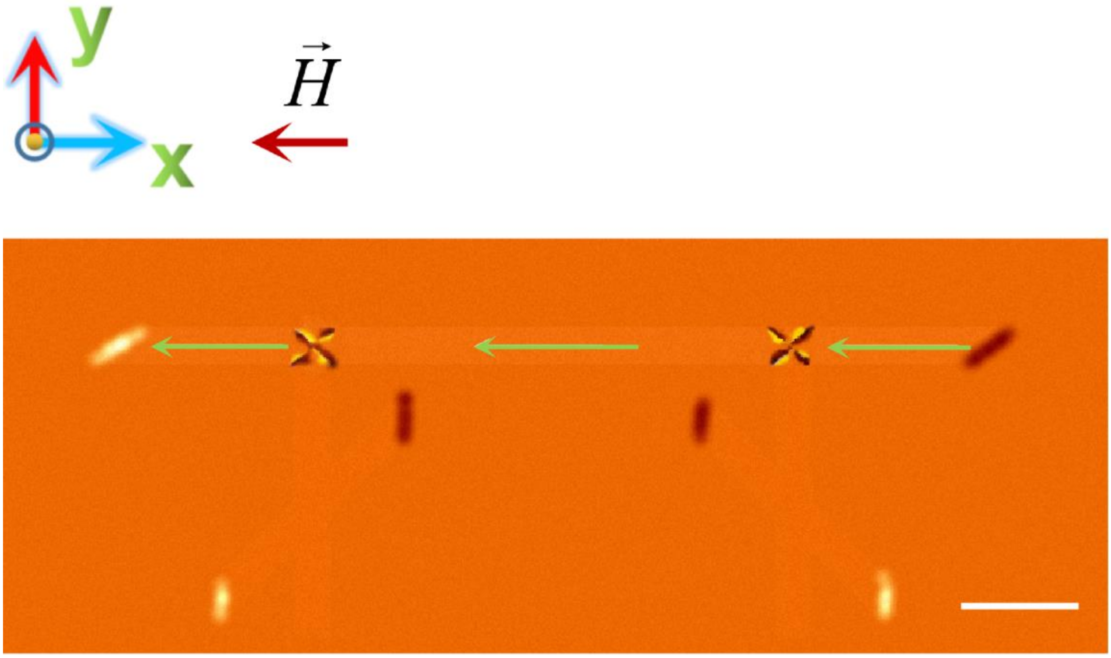

Figure S9. Verifying the stability of vortices.The green arrows indicate the magnetization directions.The white colour bar represents 1 μm.

Section 10: Verifying the stability of DVW

The experimental procedure is similar to that of Figure 2 (b)-(d) in the main text, and then H is reduced to 0, the AF-MFM image of DVW with zero magnetic field is shown in Figure S10, it can be seen that DVW is stable. The white colour bars represent 1 μm, the green arrows represent the magnetization directions.

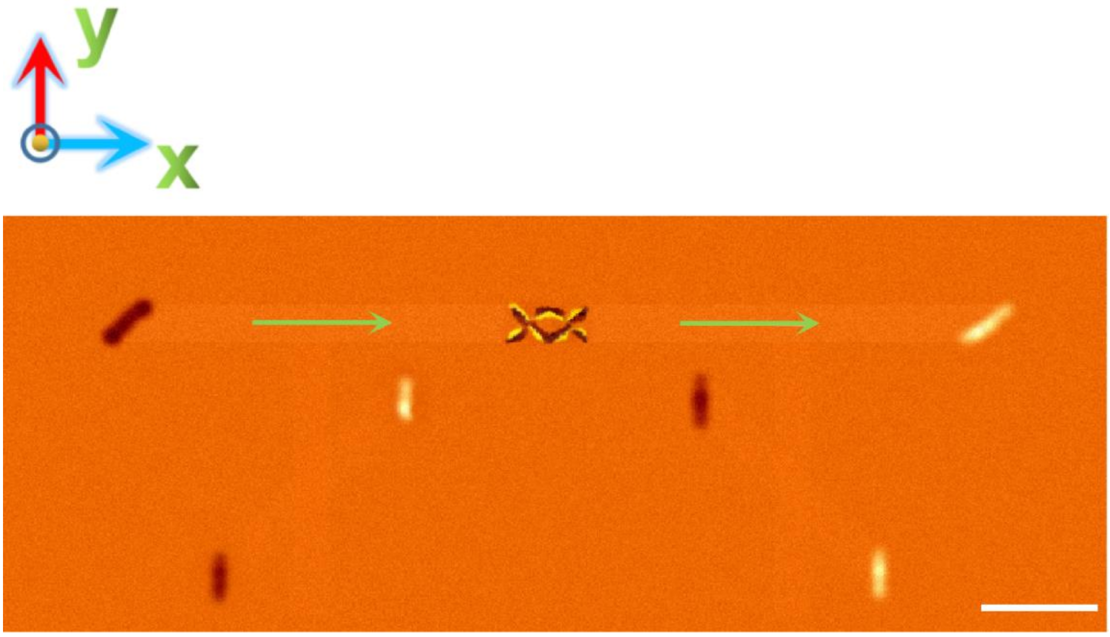

Figure S10. Verifying the stability of DVW by AF-MFM. The green arrows indicate the magnetization directions.The white colour bar represents 1 μm.

Section 11: Formation mechanism of TVW

The formation of coupled-vortex experiences the following procedure: the reversal of magnetization (-x axis to x axis, the magnetic poles at the end of nanostrip change polarity), the movement of vortex walls, the generation of vortex core, and the coupling of vortices. Therefore, we need to gradually increase the applied field H from 0 to 140 Oe to finish the magnetization reversal process, as shown in Figure S11. Figure S11 (a)-(e) show the magnetization reversal of the Fe_4N nanostrip with width of 300 nm and thickness of 30 nm. The initial magnetic configuration is set the same as measured AF-MFM image (Figure 2(h) in the main text). The magnetic field H along +x axis is configured as follows: 0 Oe→50 Oe→120 Oe→140 Oe. Figure S11 (a) shows the initial formed vortex pair (CW and CW) inside a Fe_4N nanostrip when

H =0. When H is 50 Oe along +x axis, the longitudinal domains near two tapered ends of Fe_4N nanostrip firstly reverse to +x axis, suggesting that the magnetic reversal begins by coherent magnetization rotation, as shown in Figure S11 (b). With the increase of H to 100 Oe along +x axis, the cores of the vortex pair move along opposite direction, as shown in Figure S11 (c), suggesting that the magnetic reversal shifts to domain wall motion.When H is further increased to 120 Oe along +x axis,the CW-CW vortex pair are coupled through the generation of a new vortex core at the bottom edge (marked by red circle) for energy minimization, as shown in Figure S11 (d). Further increasing H to 140 Oe, CW-CW vortex pair converge to form a triple-vortex-wall (TVW), including three vortices with a new ACW core in the center and two CW cores on both sides (Figure S11(e)). After the reversal process, the magnetic poles at the end of Fe_4N nanostrip change polarity.

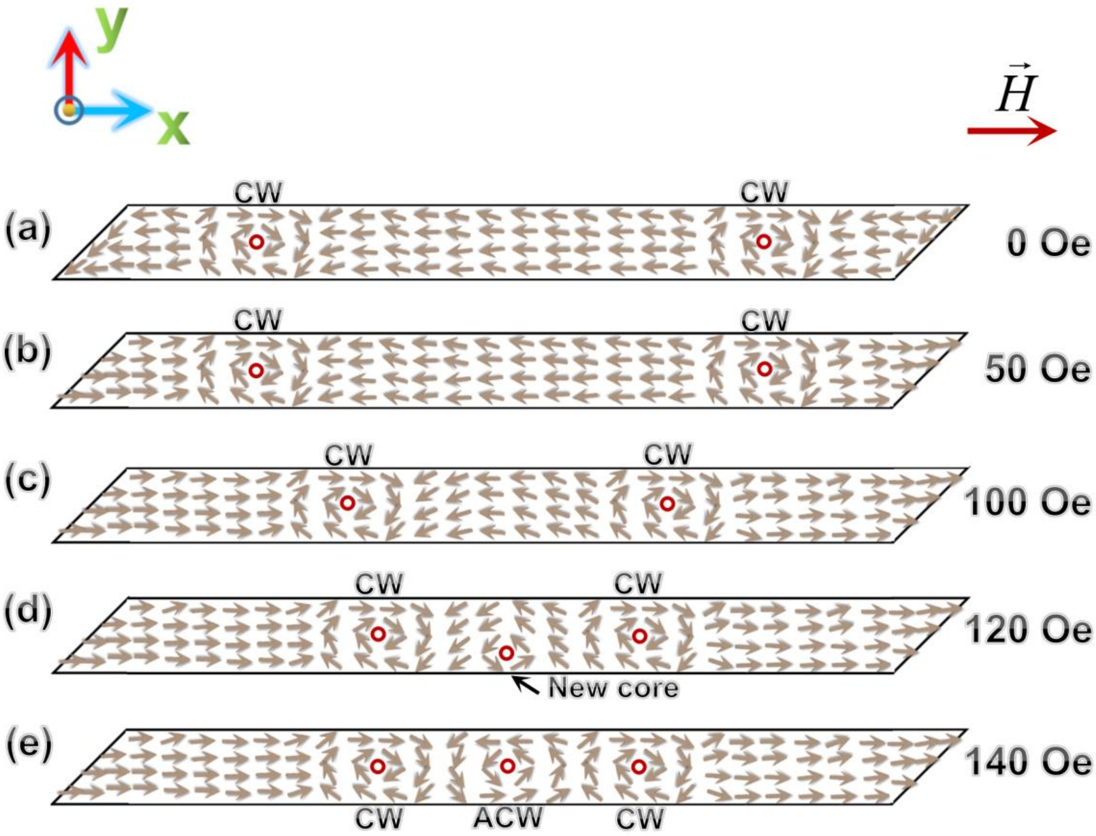

Figure S11. The formation mechanism of TVW with the increase of H from 0 to 140 Oe along +x axis.

Section 12: Verifying the stability of TVW

Figure S12 shows the AF-MFM and micromagnetic results of TVW. The experimental procedure is similar to that of Figure 2 (g)-(i) in the main text, and then H is reduced to 0, the AF-MFM image of TVW with zero magnetic field is shown in Figure S12 (a), it can be seen that TVW is stable. Figure S12 (b) shows the micromagnetic result of TVW with zero magnetic field. The formation of TVW is similar to that of Figure S11 (a)-(e), afterwords, H is reduced to 0 and the TVW keeps stable.

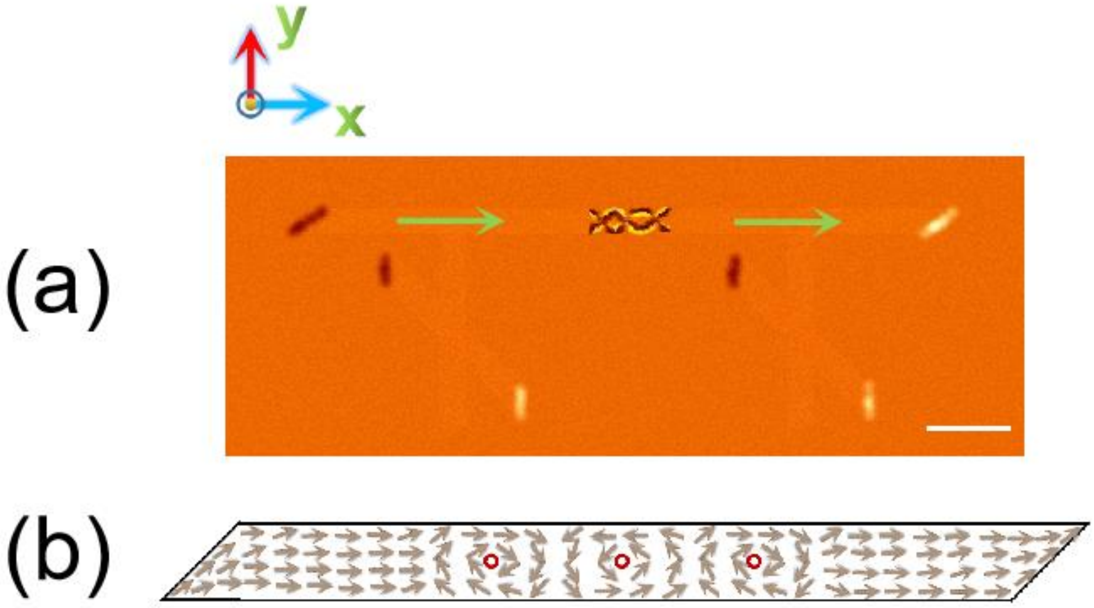

Figure S12. Verifying the stability of TVW by AF-MFM and micromagnetics. The white colour bar in (a) represents 1 μm, the green arrows in (a) represent the magnetization directions.

Section 13: Formation of five-vortex chain

The formation process of five-vortex chain is shown in Figure S13, the magnetization reversal mechanism is similar to that of Figure S11. The formation of five-vortex chain is accompanied by the reversal of magnetization of the nanostrip from -x axis to x axis (the magnetic poles at the end of nanostrip change polarity), as shown in Figure S13.

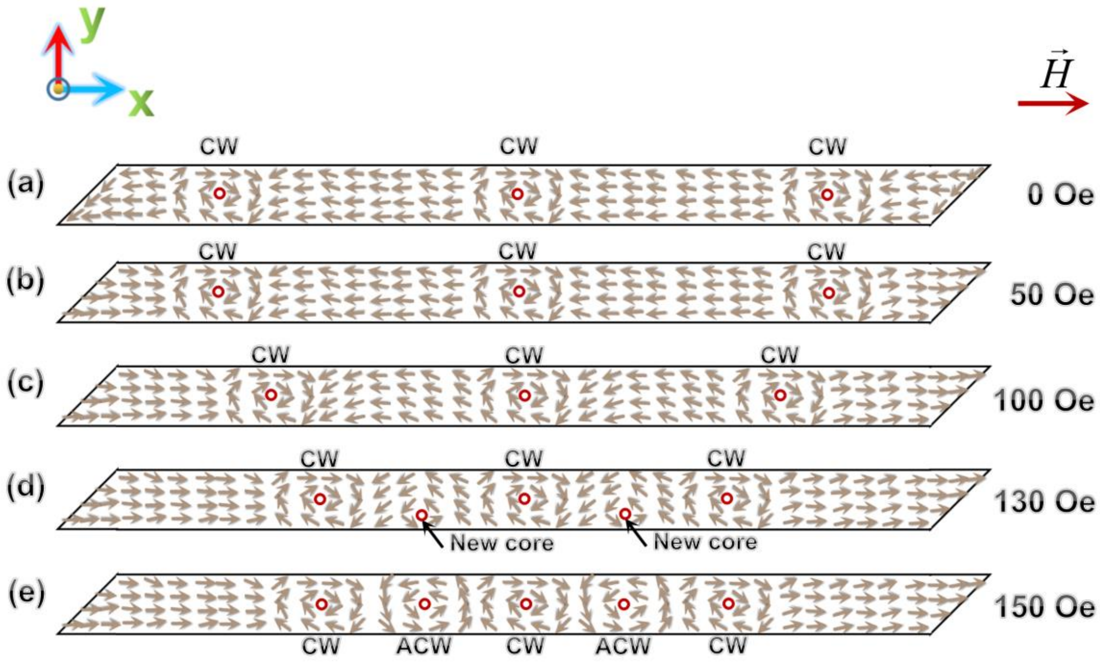

Figure S13. The formation of five-vortex chain.

Section 14: Spin wave excited in a single vortex

Figure S14 shows the mechanism of spin wave propagation along the Fe_4N nanostrip. The width and thickness of Fe_4N nanostrip are set to be 300 nm and 30 nm, respectively. A single vortex is configured at the center of nanostrip. An oscillating magnetic field h_{rf} ($h_{rf}=h_0\sin(\omega t)$) generated by microwave current is modeled for the local excitation of spin waves. The excitation frequency $f = \omega/2\pi$ is set to 1.2 GHz and 5 GHz, respectively. The amplitude of the oscillating magnetic field is around 50 Oe. The bright and dark contrasts in Figure S14 represent the calculated z-component magnetization distribution (M_z) at a certain time once a stable oscillation is achieved. At the excitation frequency of 1.2 GHz, the excited mode of spin wave shows a strong localized feature inside the vortex. However, when increasing the frequency to 5 GHz, the spin wave mode presents a periodical oscillation character along the nanostrip,

indicating the radiation of spin wave from vortex to the whole sample.

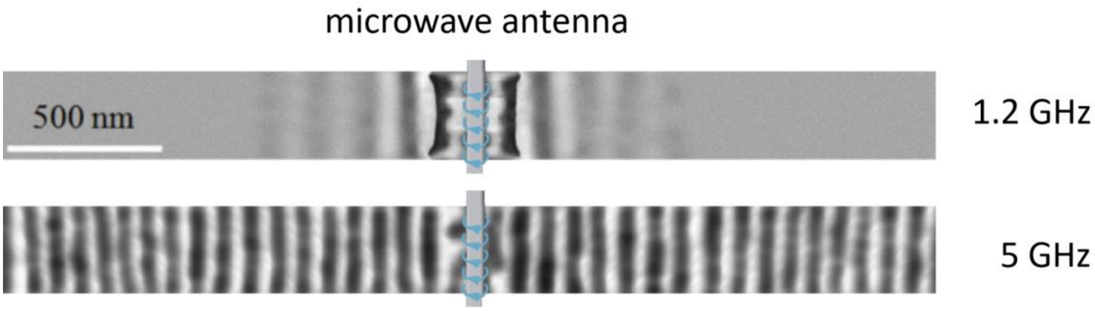

Figure S14. Perpendicular component of magnetization distribution (M_z) along the nanostrip excited by microwave fields at 1.2 and 5.0 GHz,respectively.

Section 15: Near-field Brillouin light scattering spectroscopy

The spin wave intensity was detected by near-field Brillouin light scattering spectroscopy (BSL) incorporated with an atomic force microscopy (AFM). The sample was placed onto the stage of AFM, see Figure S15. A nanosize aperture with the diameter of 40 nm was made in a hollow pyramidal AFM tip. Probing light from a single-frequency laser with the wavelength of 532 nm focused into the aperture, and interacted with the sample. The spatial resolution of the system is determined by the diameter of the aperture and tip-sample distance of AFM.

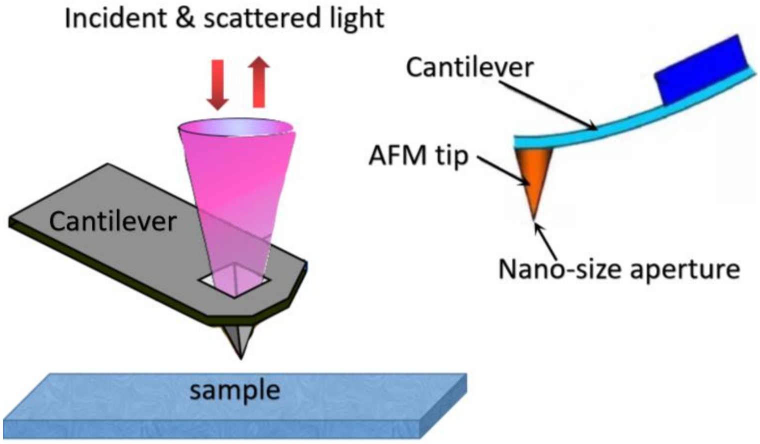

Figure S15. Near-field BSL incorporated with AFM

Section 16: SEM of the Fe_4N nanostrip incorporated with a microwave antenna

Figure S16 presents the scanning electron microscopy (SEM) image of Fe_4N nanostrip incorporated with a microwave antenna. The microwave antenna is positioned at the center (notch) of Fe_4N nanostrip. Oscillating magnetic fields h_{rf} generated by microwave currents in this antenna allow for the local excitation of spin waves with well-defined frequencies. In this work, the vortex chain is excited by h_{rf} generated by microwave current ($I_{rf}=I_0\sin(\omega t)$) of the antenna, the excitation frequency $f = \omega/2\pi$ is varied between 1 and 5 GHz with amplitudes of the current around 1 mA.

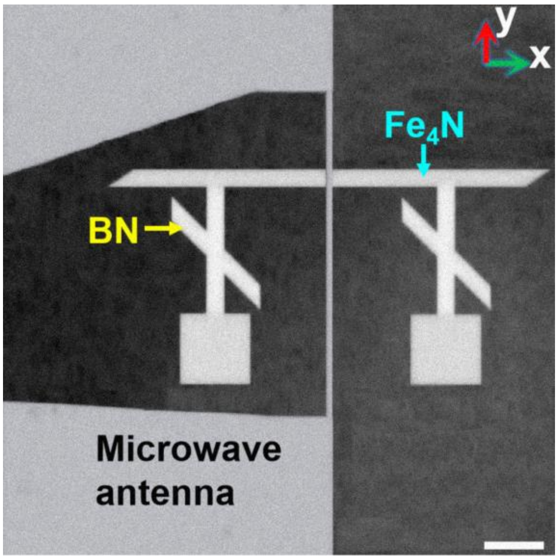

Figure S16. SEM of the Fe_4N nanostrip incorporated with a microwave antenna

Section 17: Winding number analysis of vortex reversal from ACW to CW

The chirality switching of vortex is governed by the conservation of topological

defects, the analysis of the winding number is shown in Figure S17. The simulated dimensions of Fe_4N nanostrip and BN are accorded with experiments. The chirality reversal of vortex from ACW to CW are shown for 45° cross-type nanostructure. The vortex with ACW chirality is injected into Fe_4N nanostrip and parked before BN, as seen in Figure S17(a). The vortex moves toward the BN, and gradually contracts its size, as shown in Figure S17 (b). The initial ACW core annihilate when an extra vortex with CW chirality nucleates near the cross point of Fe_4N and BN, as seen in Figure S17 (c). The CW vortex expands its size (Figure S17 (d)) and finally escapes from BN (Figure S17 (e)) . Notably, the topological defects of the system is conserved as the vortex passing across the BN.

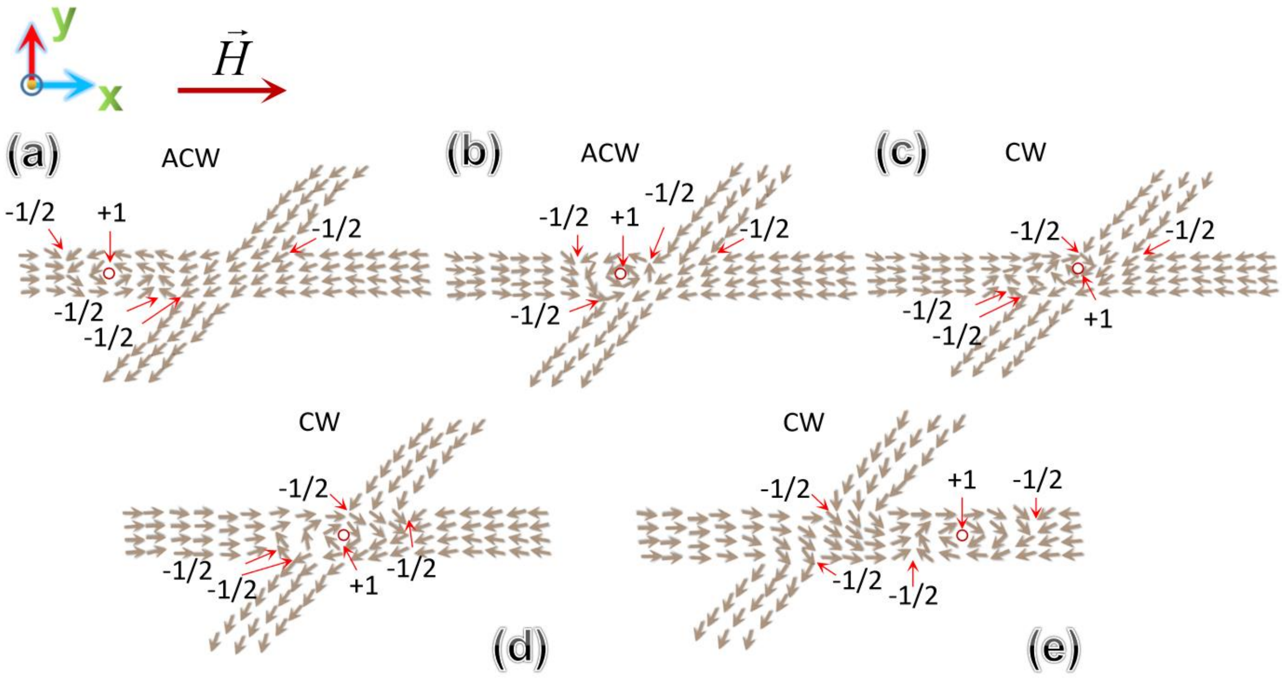

Figure S17. The chirality reversal of vortex from ACW to CW for 45° cross-type nanostructure, the winding number is marked.

Section 18: Chirality switching of vortex in 45° cross-type nanostructure

A micromagnetic model is established to demonstrate the chirality switching process of vortex in 45° cross-type nanostructure with Fe_4N nanostrip and BN. In the micromagnetic simulation (OOMMF program, code NIST.18), the energy terms include the Zeeman, crystalline anisotropies, shape anisotropies, magnetic exchanges, and magnetostatic energies. Standard parameters of Fe_4N materials are selected: saturation magnetization of 1.4 MA/m, crystalline anisotropy constant of 3.0×10^4 J/m^3, exchange stiffness of 12.5 pJ/m, and damping coefficient of 0.5. The reversals of magnetic moments obey the law of Landau–Lifshitz–Gilbert (LLG) equations.

Figures S18 (a)-(h) present the vortex evolution process in Fe_4N nanostrip with width of 300 nm and thickness of 30 nm. Fe_4N nanostrip is initially magnetized by H of 1000 Oe along -x axis, and then H shifts to +x axis and configures as follows: 50 Oe→75 Oe→100 Oe→150 Oe. When H=50 Oe, the vortices with both CW and ACW chiralities are injected into Fe_4N nanostrip and parked before BN during the magnetic equilibrium process, as seen in Figure S18 (a) and (e). With the increase of H to 75 Oe, the vortices move along the nanostrip and approach BN, as shown in Figure S18 (b) and (f). when H is further increased to 100 Oe, the CW core keeps stable and parks at the centre of BN (Figure S18 (c)), meanwhile, the initial ACW core gradually contracts its size and will annihilate near top of Fe_4N nanostrip (red arrow in Figure S18 (g)). When H=150 Oe, the CW core move through BN without changing the chirality (Figure S18 (d)), however, the ACW core change to CW after passing through the BN (Figure S18 (h)). Figures S18 (i)-(l) show the calculated MFM images

related to Figures S18 (e)-(h) , the simulated results accord with experiments.

As a single vortex approaches BN, if the spins near the guiding edge of vortex oriented parallel to the magnetization inside BN (green arrow in Figure S18 (b)), the vortex can pass through the barrier and conserved its chirality for energy minimization. Contrarily, when the spins near the guiding edge of vortex oriented anti-parallel to the magnetization of BN (green arrow in Figure S18 (f)), the chirality will reverse after vortex passing through the barrier to avoid the magnetic charge accumulation.

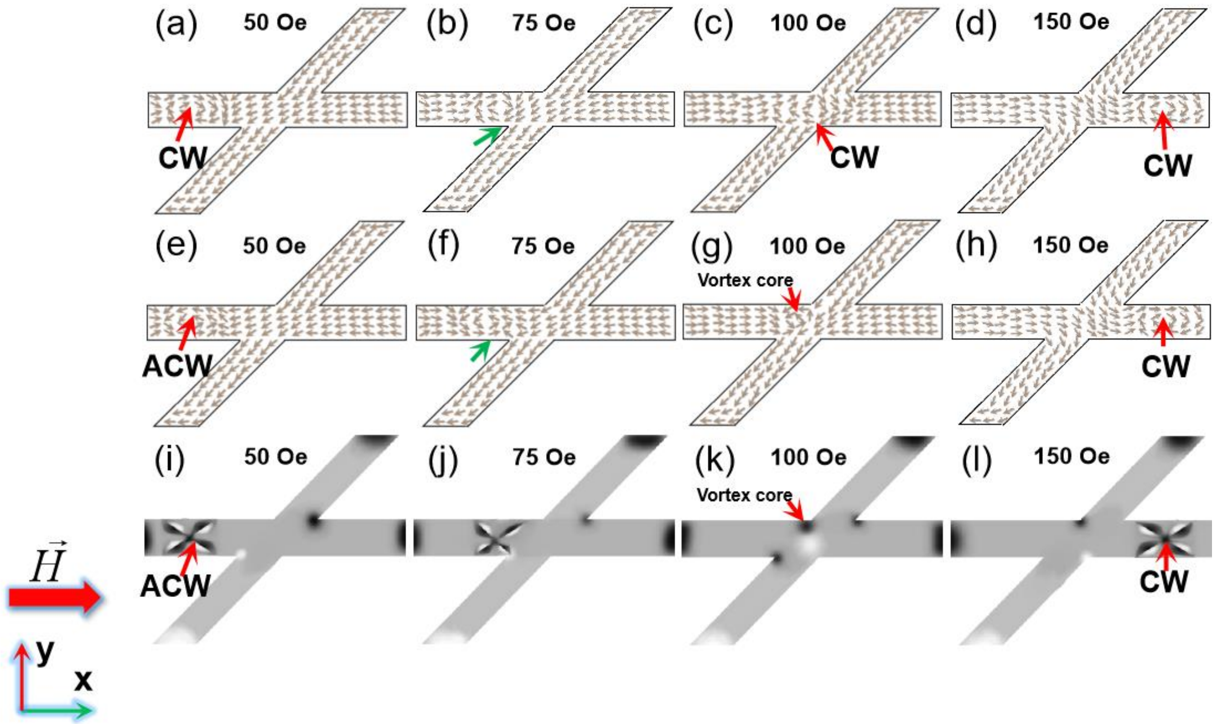

Figure S18. (a-h) Chirality switching of vortex in a 45° cross-type nanostructure with Fe_4N nanostrip and BN (i-l) calculated MFM images related to (e)-(h).

Section 19:Principles of AF-MFM technique

Figure S19 shows fundamental principles of AF-MFM by lock-in technique for detecting sample magnetization. An AC voltage V(ω) is applied to ferrite core (the magnetic ferrite core are composed of Fe_2O_3 (55%), NiO (20%) and ZnO (25 %)) to produce a modulated H-field perpendicular to film plane, which can periodically transform magnetization directions in magnetic samples, and generate an AC magnetic field B(ω) from sample surface. B(ω) is detected by a hard MFM tip lifted above the sample surface, generating a frequency-modulated MFM signal A (ω) (frequency modulation of cantilever resonance) which is extracted using a phase-locked-loop. Demodulated signal C(ω) is then fed into lock-in amplifier to produce measured MFM signal. Since AF-MFM technique is based on a frequency modulation (FM) of the cantilever oscillation, the modulated MFM signal decays with the increase of frequency. When the frequency reaches 10 KHz, the MFM signal is too weak to be detected, therefore, the maximum measured frequency is limited to 10 KHz.

In AF-MFM, resonant frequency (f_0) of cantilever is 256 kHz. AC voltage and frequency is 0.2 V and 125 Hz, respectively. AFM/MFM images are captured under lift/tapping modes (the lift height of 2-5 nm) using a high coercivity FePt tip. The FePt (20 nm)/MgO(5 nm) films were deposited on AFM Si tips (spring coefficient of 40 N/m) at room temperature using magnetron sputtering technique. During the film growth, the sputtering pressure of Ar gas was set to 3.9 mTorr, and the power of sputtering was fixed at 200 W. Afterwards, the FePt (20 nm)/MgO(5 nm) films were annealed at 450 °C for 1.5 h. The FePt tip can get closer to material surface without

admixing the atomic phase, therefore, spatial resolution of AF-MFM can be better than 5 nm.

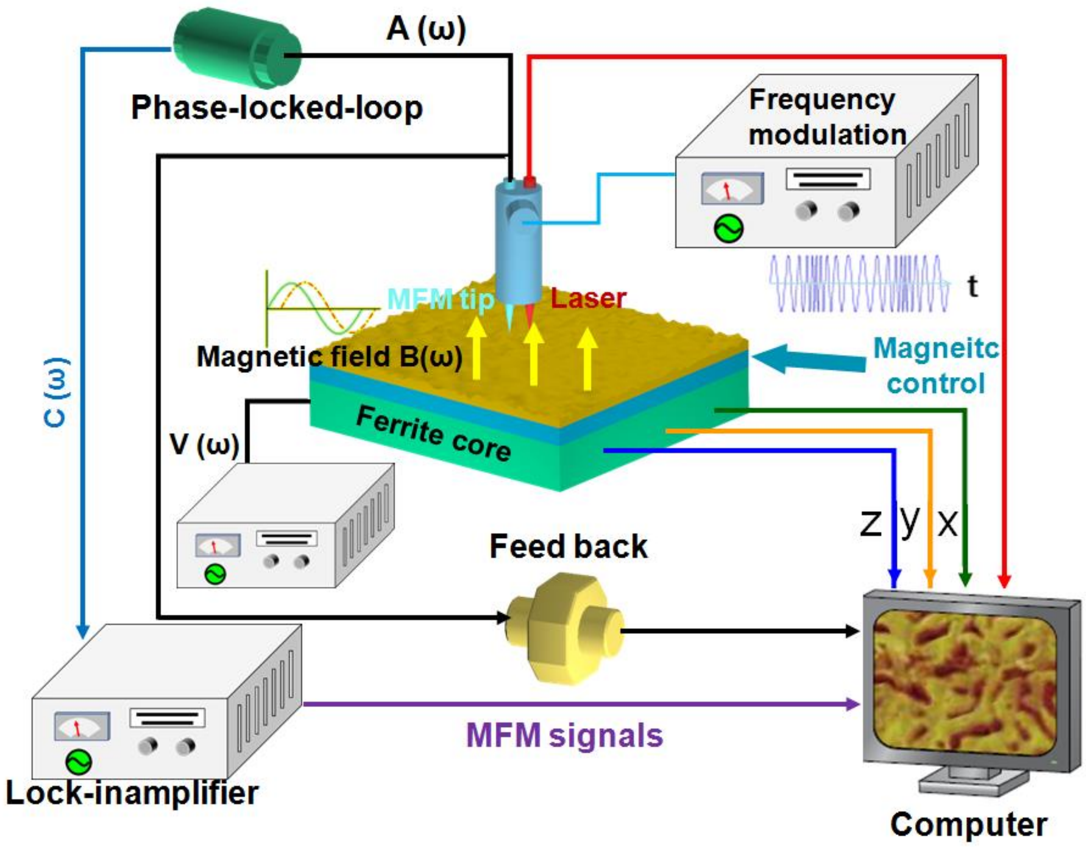

Figure S19 . The fundamental principles of the AF-MFM technique.

In the AF-MFM, the MFM tip oscillations can be described as

$$m\frac{d^2z(t)}{dt^2}+m\gamma\frac{dz(t)}{dt}+(k_0+\Delta k)z(t)=F_0\cos(\omega_0 t) \qquad \text{(S1)}$$

here z , γ , m and k_0 are the displacement, damping constant, effective mass and intrinsic stiffness of the oscillated MFM tip. Δk is the effective stiffness factor of the MFM tip, and $F_0\cos(\omega_0 t)$ is an alternating force from piezo-electric elements.

An AC voltage $V(\omega_m)$ is applied to the ferrite core to produce a modulated

H-field perpendicular to sample surface, which can periodically transform the magnetization directions in magnetic samples. The magnetization $M^{ac}_{sample}(t)$ of samples rotates periodically,

$$M^{ac}_{sample}(t) = M_z(t) + M_x(t) = M^{ac}_z \cos(\omega_m t) + M^{ac}_x \sin(\omega_m t) \qquad \text{(S2)}$$

here M^{ac}_z, M^{ac}_x are the components of magnetization perpendicular and parallel to sample surface, respectively.

The AF-MFM images are imaged under lift /tapping modes using a high coercivity (more than 10 KOe) $L1_0$-FePt tip, the MFM tip can be treated as a magnetic monopole, the effective stiffness factor of the MFM tip is defined as

$$\begin{aligned}
\Delta k(t) &\cong q^{dc}_{tip} \frac{\partial H^{ac}_z (M^{ac}_{sample}(t))}{\partial z} \\
&= q^{dc}_{tip} \left(\frac{\partial H^{ac}_z (M^{ac}_z \cos(\omega_m t))}{\partial z} + \frac{\partial H^{ac}_z (M^{ac}_x \sin(\omega_m t))}{\partial z} \right) \\
&\cong q^{dc}_{tip} \left(\frac{\partial H^{ac}_z (M^{ac}_z)}{\partial z} \cos(\omega_m t) + \frac{\partial H^{ac}_z (M^{ac}_x)}{\partial z} \sin(\omega_m t) \right) \\
&= \Delta k_0 \cos(\omega_m t + \phi)
\end{aligned} \qquad \text{(S3)}$$

here q^{dc}_{tip} is the effective magnetic monopole, H^{ac}_Z is the alternating magnetic field from sample surface, and ω_m is the frequency of AC voltage.

Base on lock-in technique, the in-phase (X) and out-of-phase (Y) signals, corresponding to the sine and cosine parts in equation (S3), can be extracted as

$$X + iY \propto \frac{\partial H^{ac}_z (M^{ac}_z)}{\partial z} \cos(\omega_m t) + i \frac{\partial H^{ac}_z (M^{ac}_x)}{\partial z} \sin(\omega_m t) \qquad \text{(S4)}$$

Meanwhile, the amplitude (A) and phase (P) signals can also be extracted by lock-in technique:

$$A = \left\{ \left[\frac{\partial H_z^{ac}(M_z^{ac})}{\partial z} \right]^2 + \left[\frac{\partial H_z^{ac}(M_x^{ac})}{\partial z} \right]^2 \right\}^{1/2} \quad \text{(S5)}$$

$$P = \arctan \left\{ \frac{\partial H_z^{ac}(M_x^{ac})}{\partial z} \middle/ \frac{\partial H_z^{ac}(M_z^{ac})}{\partial z} \right\} \quad \text{(S6)}$$

Section 20: Description of the micromagnetic theory

In the micromagnetic simulation (OOMMF program, code NIST.18), standard parameters of Fe_4N materials are selected: saturation magnetization of 1.4 MA/m, crystalline anisotropy constant of 3.0×10^4 J/m^3, exchange stiffness of 12.5 pJ/m, and damping coefficient of 0.5. The energy terms include Zeeman, crystalline anisotropies, shape anisotropies, magnetic exchanges, and magnetostatic energies:

$$E^i_{total} = E^i_{Zee} + E^i_{ck} + + E^i_{sk} + E^i_{ex} + E^i_m \quad \text{(S7)}$$

The effective field $\vec{H}^i_{eff}$ is defined as :

$$\vec{H}^i_{eff} = -\frac{1}{\mu_0}\frac{\partial E^i}{\partial \vec{M}^i} \quad \text{(S8)}$$

Where $\vec{M}^i$ is the magnetization in a grid.

The reversals of magnetic moments obey the law of Landau–Lifshitz–Gilbert (LLG) equations:

$$\frac{d\vec{M}^i}{dt} = -\gamma \vec{M}^i \times \vec{H}^i_{eff} - \frac{\alpha}{M_s}\vec{M}^i \times (\vec{M}^i \times \vec{H}^i_{eff}) \quad \text{(S9)}$$

where Ms , γ and α present the saturation magnetization, gyromagnetic coefficient, and damping constant, respectively.
